# A Customizable Quantum-Dot Cellular Automata Building Block for the Synthesis of Classical and Reversible Circuits

**DOI:** 10.1155/2015/705056

**Published:** 2015-08-09

**Authors:** Ahmed Moustafa, Ahmed Younes, Yasser F. Hassan

**Affiliations:** ^1^Department of Mathematics and Computer Science, Faculty of Science, Alexandria University, Alexandria 21511, Egypt; ^2^School of Computer Science, University of Birmingham, Birmingham B15 2TT, UK

## Abstract

Quantum-dot cellular automata (QCA) are nanoscale digital logic constructs that use electrons in arrays of quantum dots to carry out binary operations. In this paper, a basic building block for QCA will be proposed. The proposed basic building block can be customized to implement classical gates, such as XOR and XNOR gates, and reversible gates, such as CNOT and Toffoli gates, with less cell count and/or better latency than other proposed designs.

## 1. Introduction

One of the challenging problems in the development of computation paradigms and systems is data loss [1]. Reversible computation is a possible solution to solve this problem by allowing the computation to be done at the logical level without data loss by establishing a one-to-one and onto mapping (bijection) between the inputs and outputs of the circuit, that is, the number of inputs equal to the number of outputs [1]. Efforts have been done in exploring the capabilities of emerging technologies to perform reversible computation.

A quantum-dot cellular automaton (QCA) is a promising emerging technology that works on novel paradigms such as synthesis of reversible gates [2]. QCA is a constructing nanoelectronic technology that gives another approach to computation at nano level [3]. Research and development in the field of electronic devices during the last decades made it possible for designers to increase the speed and decrease the size of the components and the power consumption. QCA is based upon the encoding of binary information in the electron charge configuration within quantum-dot cells. Computational power is provided by the Coulombic interaction between QCA cells. There is no current flow between cells and no outer source is delivered to singular internal cells [4]. Due to the reordering of electron positions, the physics of cell-to-cell interaction provides the local interconnections between cells [5, 6]. Lent and Tougaw in 1993 introduced the basic concepts of QCA [3, 7] as the computation with cellular automata consists of arrays of quantum-dot cells. The unique feature is that logic states are represented by a cell. A cell is a nanoscale device able to encode data by two-electron configuration. The cells must be aligned exactly at nanoscales to provide correct functionality; thus, the testing of these devices for misalignment and manufacturing errors has an important role for the correctness of circuits [8]. The relation between computation and data loss has been solved in QCA because it has very low power consumption which is a common property for QCA [9–11]. In [12–14], different QCA designs for XOR gate have been shown. In [2, 15, 16], designs for Toffoli gate have been proposed.

The aim of this paper is to propose QCA designs for classical and reversible gates using a basic building block. The basic building block can be customized to implement the required gate. Based on the proposed building block, QCA designs for classical gates such as XOR and XNOR will be proposed. Customization of the proposed building block makes it possible to extend the proposed design of the XOR gate to implement reversible gates such as CNOT and Toffoli gates. Moreover, the basic building block can be used to implement reversible circuits that contain more than one reversible gate. The proposed QCA circuits in this paper have been designed and simulated using the QCADesigner tool version 2.0.3 running the system with coherence simulation engine [17].

This paper is organized as follows. A literature review is presented in Section 2. A review of the QCA basics and clocking is given in Section 3. The proposed QCA for the XOR gate and reversible gates is shown in Section 4. Finally Section 5 concludes the paper.

## 2. Literature Review

The design of QCA for reversible functions is gaining attention in the literature. Many designs for the XOR gate using QCA have been proposed. In 2012, Ahmad and Bhat [12] proposed two different designs for the XOR gate. The first design consists of 37 cells. It uses three MV (Majority Voting) gates; one MV gate is used as OR gate, while the second and third MV gates are used as AND gate. The second design has crossover and it consists of 30 cells; this design uses the same number of MV gates as in the first design. Both designs have 0.5 clock delay. In 2013, Beigh et al. [13] proposed seven different designs for the XOR gate. The first and seventh designs are the most efficient designs among the seven designs in terms of cell count and clock delay. The first design consists of 34 cells and has 1 clock delay. It uses four MV gates; one MV gate is used as OR gate, while the second, the third, and the fourth MV gates are used as AND gate. The seventh proposed design consists of 42 cells and has 0.5 clock delay. It uses three MV gates; one is used as OR gate, while the second and third MV gates are used as AND gate. In 2014, Santra and Roy [14] proposed a design of the XOR gate that consists of 30 cells and has 4 clock delay. It uses three MV gates; one is used as OR gate while the second and third MV gates are used as AND gate.

Many designs for Toffoli gate using QCA have been proposed. In 2008, Ma et al. [2] proposed QCA design of Toffoli gates that consists of 169 cells and has 4 clock delay. This design uses four MV gates. It is a complicated design due to the large number of cell count. In 2013, Bahar et al. [15] proposed two different designs for the Toffoli gate. The first design consists of 75 cells and has 1 clock delay. This design uses four MV gates; three MV gates are used as AND gate while the fourth MV gate is used as OR gate, while the second design consists of 48 cells with 1 clock delay, where it uses four MV gates; three MV gates are used as AND gate while the fourth MV gate is used as OR gate similar to their first design. In 2013, Rolih [16] proposed another design for the Toffoli gate using smaller number of cell count where the QCA design for the Toffoli gate consists of 44 cells and has 1 clock delay.

## 3. QCA Basics

Before addressing the proposed layouts, it is important to review the basic properties of QCA. This section gives a brief overview of the device physics, basic logic gates, and clocking.

### 3.1. Basic Quantum-Dot Construction

**Figure 1:**
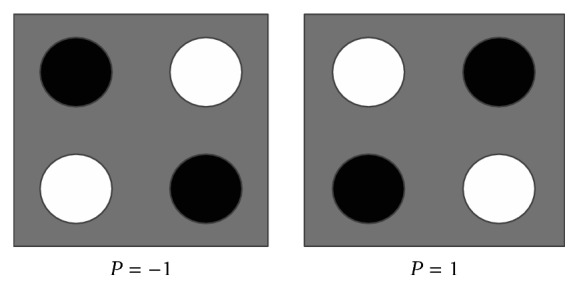
QCA cell polarization.

The computational elements and wires of an entire circuit are built through QCA devices. The basic component of a QCA device is a cell with four quantum dots placed in the corners and two free electrons. By applying sufficient local electric field on the tunneling junctions, the potential barriers control the transition of the mobile electrons to provide three states for the isolated cell. The first state is* null cell* which occurs when the barriers are lowered by decreasing the electric field; this allows electrons to be found on any dots. The second state is* positive polarization* (*P* = +1) which occurs when the barriers are raised positively. The third state is* negative polarization* (*P* = −1) that occurs when the barriers are raised negatively. The terms* positive polarization* and* negative polarization* are corresponding to binary logic values “1” and “0,” respectively.  Figure 1 shows the basic QCA cells and their possible polarizations. Coulombic interactions between the cells are placed near each other to be forced into matching polarizations. More details for the QCA device physics can be found in [3].

### 3.2. QCA Wires

**Figure 2:**
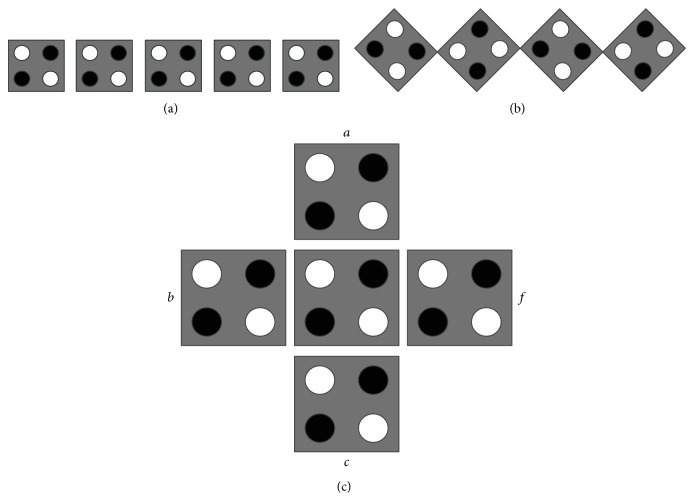
(a) QCA wire (90°), (b) QCA wire (45°), and (c) QCA majority gate.

In a QCA wire, the binary information moves from input to output because of the Coulombic interactions between cells. The propagation in a 90° QCA wire is shown in Figure 2(a). A 45° QCA wire, as shown in Figure 2(b), is also possible where the propagation of the binary signal alternates between the two different polarizations +1 and −1 [18].

### 3.3. QCA Majority Gate and Inverter

**Figure 3:**
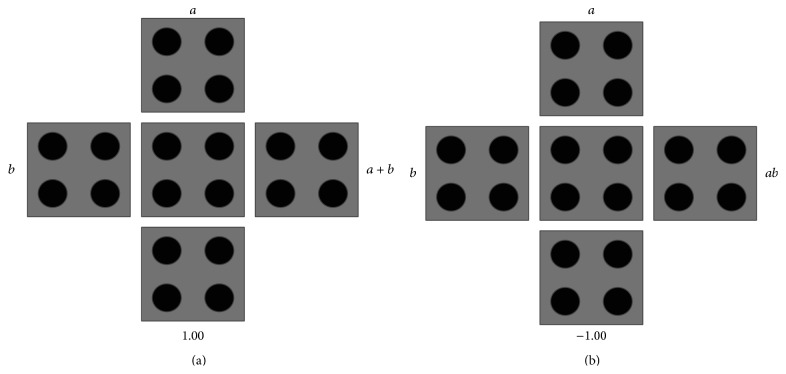
QCA layout of (a) OR gate and (b) AND gate.

**Figure 4:**
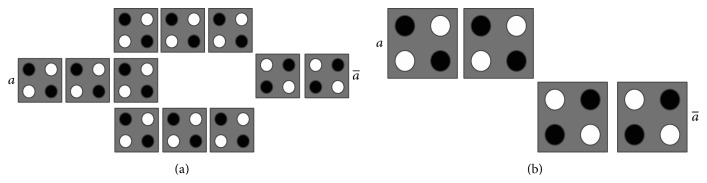
QCA inverters.

**Figure 5:**
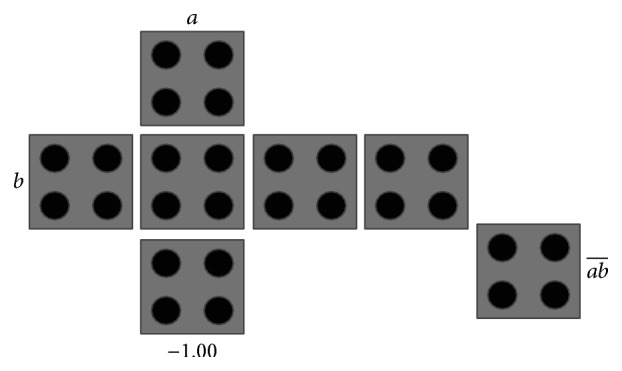
QCA layout of NAND gate.

The main QCA logical circuit is the three-input MV gate as shown in Figure 2(c). The MV gate consists of five QCA cells, three input cells, one device cell, and one output cell [17]. Suppose that the three inputs are *a*, *b*, and *c*; then the logic function of the MV gate is *M*(*a*, *b*, *c*) = *ab* + *bc* + *ac*, where *a* + *b* denotes *a* OR *b* and *ab* denotes *a* AND *b*. By using the MV gate, we can construct the OR and the AND gates by fixing the polarization of one of the inputs of the MV gate to *P* = +1 (logic “1”) and *P* = −1 (logic “0”) as shown in Figures 3(a) and 3(b), respectively. Another popular QCA gate is the NOT gate or the inverter. One construction of QCA inverter is shown in Figure 4(a). Another construction of the NOT gate is shown in Figure 4(b), where the input is inverted due to the different polarizations that are misaligned between the touching corners of the cells [19]. The NAND gate can be constructed using the AND gate followed by the NOT gate as shown in Figure 5.

### 3.4. QCA Clocking

**Figure 6:**
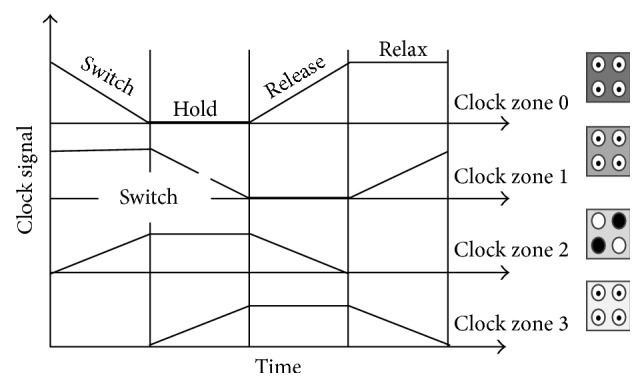
The four phases of the QCA clocking.

Synchronization and control of information flow are provided by QCA clocking to give the power to run the QCA circuit because there is no outer source for that purpose. All the proposed designs in this paper have been simulated using coherence simulation engine with clock high = 9.8*e* − 22 J and clock low = 3.8*e* − 23 J [17]. The QCA clocking is achieved by managing the potential barriers between adjacent quantum dots. Controlling the potential barrier by raising or lowering allows full control of localization and polarization for the mobile electrons as follows: raising potential force elements to localize and hence definite polarization occur while the lowering potential allows delocalizing electrons and providing no definite cell polarization. The cells can be grouped into four zones to have the same electric field influence to achieve proper clocking scheme. These four zones are as follows:* switch zone* occurs when the tunneling barriers are raised causing the electrons to be effected by Columbic charges of neighboring zones. The* hold zone* has high tunneling barrier and will not change the state but influence adjacent zones. The* release* and* relax* zones occur by decreasing the tunneling barrier, so, other zones will not get influenced by that zone as shown in Figure 6. It is important to note that the size of these zones must be within certain limits, but their shape may be irregular [18]. The accurate placement of these zones is important for the efficiency of the design.

## 4. The Proposed QCA Circuits

**Figure 7:**
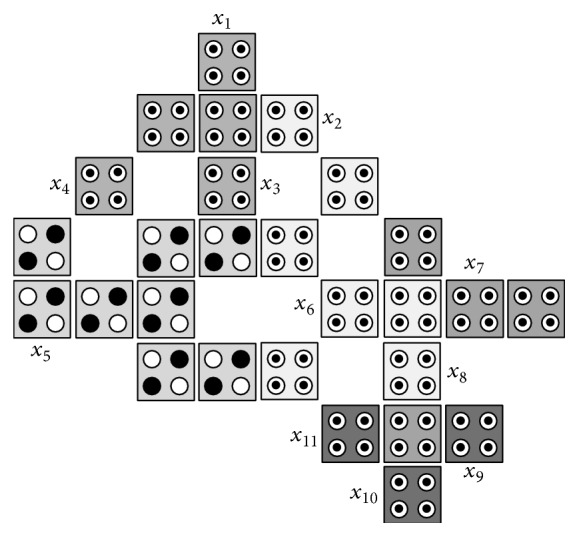
The proposed basic building block where the customization cells are *x*_1_, *x*_2_,…, *x*_11_.

We propose a basic building block as shown in Figure 7. The basic building block consists of two MV gates and NOT gate. One of the MV gates functions as AND gate, while the other functions as OR gate. The basic building block contains 27 cells, 11 of them will function as configuration cells and will be denoted as *x*_1_, *x*_2_,…, *x*_11_, where these configuration cells will be set as input cells, output cells, device cells, rotate cells, or control cells to customize the basic building block to different designs as will be shown later. The AND gate, OR gate, and NOT gate can be obtained directly from the basic building block by setting the configuration cells as shown in Table 1. Other logic gates can be obtained from the basic building block by setting up the configuration cells and/or adding extra cells as shown in Table 1.

### 4.1. QCA for Logic Gates

**Figure 8:**
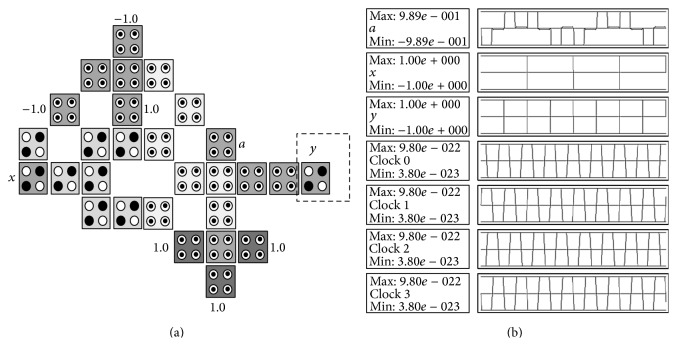
The proposed QCA for the XOR gate: (a) the layout where the added cells to the basic building block are highlighted with dashed line, (b) the simulation results.

**Figure 9:**
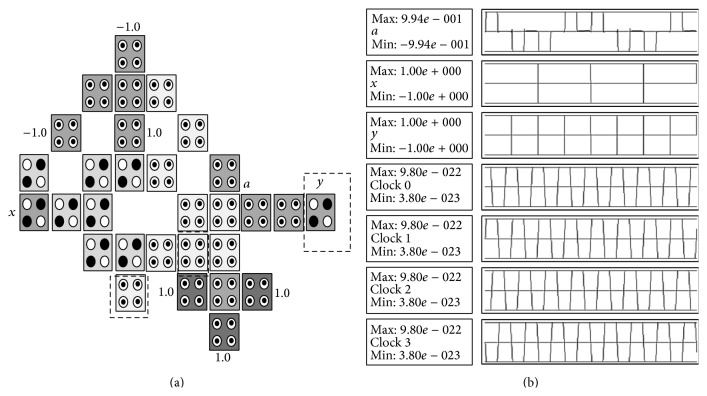
The proposed QCA for the XNOR gate: (a) the layout where the added cells to the basic building block are highlighted with dashed line, (b) the simulation results.

The XOR gate, also known as exclusive disjunction, is a logical operation on two operands that results in a logical value of true if and only if one of the operands, but not both, has a value of true and it will be denoted by ⊕. The XOR gate has the following logic operation: $a=x⊕y=\bar{x}y+\bar{y}x$, where *x*, *y* are inputs, *a* is output, and $\bar{x}$ denotes NOT *x*. The basic building block can be customized to implement the XOR gate by adding 1 cell (denoted *y*) as shown in Figure 8 where the configuration cells of the basic building block are as shown in Table 1.  Table 2 gives the comparison of the proposed design with other XOR designs. It shows that the proposed design is more efficient in terms of cell count, latency, and number of MV gates. It is important to notice that the proposed layout has neither crossover cells nor multilayers which is an advantage over the other designs shown in Table 2. The proposed layout can be used to design complex circuits based on XOR operation such as the CNOT and the Toffoli gate as will be shown later. The basic building block can be customized to implement the XNOR gate by adding 2 cells to the XOR gate as shown in Figure 9 where the configuration cells of the basic building block are as shown in Table 1.

### 4.2. QCA for Reversible Gates

**Figure 10:**
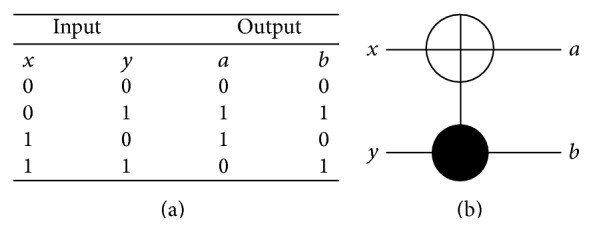
The CNOT gate: (a) truth table, (b) reversible circuit representation.

**Figure 11:**
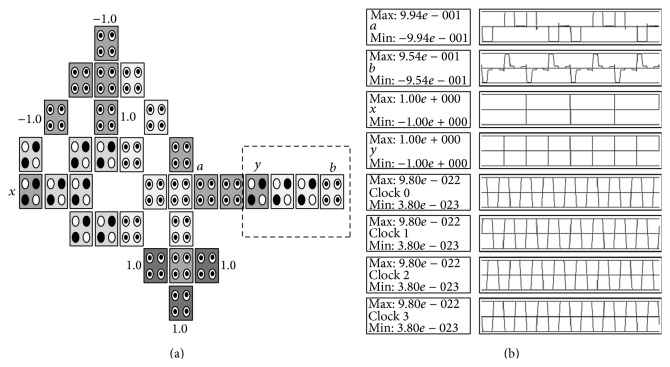
The proposed QCA for the CNOT gate: (a) the layout where the added cells to the basic building block are highlighted with dashed line, (b) the simulation results.

**Figure 12:**
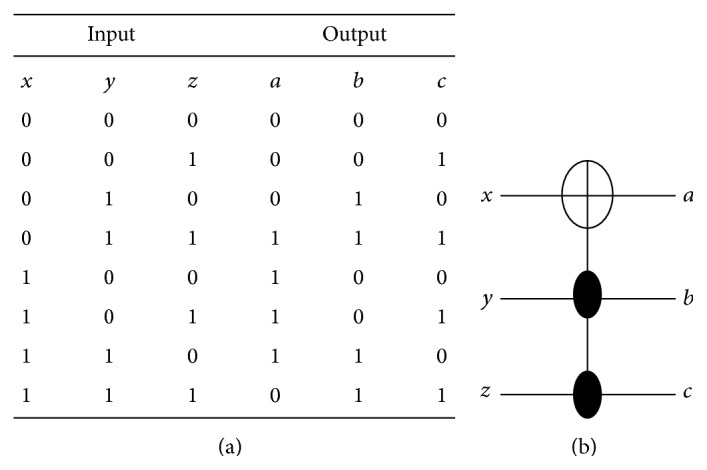
The Toffoli gate: (a) truth table, (b) reversible circuit representation.

**Figure 13:**
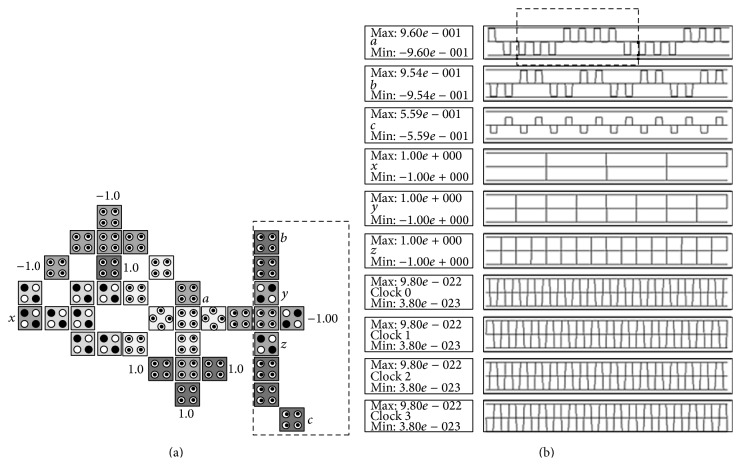
The proposed QCA for the Toffoli gate: (a) the layout where the added cells to the basic building block are highlighted with dashed line, (b) the simulation results.

The CNOT gate can be described as follows: *a* = *x* ⊕ *y* and *b* = *y* where *x*, *y* are inputs and *a*, *b* are outputs. The truth table of the CNOT gate is shown in Figure 10(a) and its reversible gate representation is shown in Figure 10(b). The basic building block can be customized to implement the CNOT gate by adding 4 cells as shown in Figure 11 where the configuration cells of the basic building block are as shown in Table 1. The CNOT gate contains two MV implementations similar to the XOR gate where it has two outputs instead of one output as in the XOR gate. The proposed CNOT gate can be generalized to implement the Toffoli gate. The Toffoli gate is a universal reversible logic gate proposed by Toffoli [20] and can be used in the construction of a reversible version of any classical gate. The Toffoli gate has the following logic operation: *a* = *x* ⊕ *yz*, *b* = *y*, and *c* = *z* where *x*, *y*, *z* are inputs and *a*, *b*, *c* are outputs. The truth table of the Toffoli gate is shown in Figure 12(a) and its reversible gate representation is shown in Figure 12(b). If both the second and third inputs are equal to logic “1” then the Toffoli gate negates its first input; otherwise the first input remains unchanged [21]. The basic building block can be customized to implement the Toffoli gate by adding 9 cells as shown in Figure 13 where the configuration cells of the basic building block are as shown in Table 1. The proposed design contains three MV gates: two MV gates are used as AND gates, while the third is used as OR gate.  Table 3 compares the proposed design with other QCA designs for the Toffoli gate. It shows that the proposed design is more efficient in terms of cell count but with two-clock-cycle delay.

**Figure 14:**
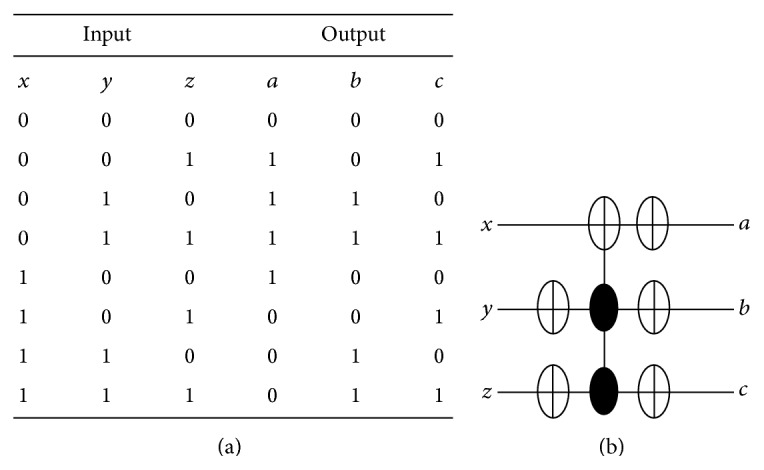
A reversible circuit with single Toffoli gate and five NOT gates: (a) truth table, (b) reversible circuit representation.

**Figure 15:**
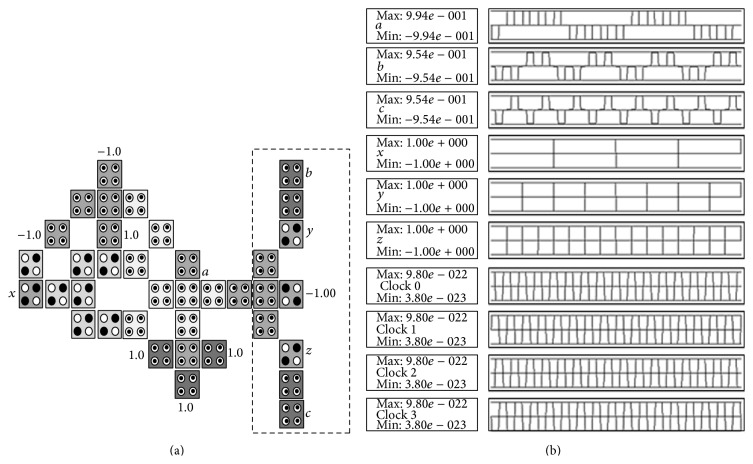
The proposed QCA for the reversible circuit shown in Figure 14: (a) the layout where the added cells to the basic building block are highlighted with dashed line, (b) the simulation results.

The basic building block can be used to implement reversible circuits. For example, consider a reversible circuit that consists of a single Toffoli gate and five NOT gates; the truth table of this reversible circuit is shown in Figure 14(a), while its circuit representation is shown in Figure 14(b). The function of this reversible circuit can be also described as follows: $a=\bar{x}⊕\bar{y}\bar{z}$, *b* = *y*, and *c* = *z*. The basic building block can be customized to implement this reversible circuit by adding 10 cells with no clock cycle delay as shown in Figure 15 where the configuration cells of the basic building block are as shown in Table 1.

## 5. Conclusion

In this paper, a basic building block that consists of two MV gates and NOT gate has been proposed; one MV gate functions as AND gate, while the other functions as OR gate with a total of 27 cells. The proposed basic building block has been used to implement classical gates such as XOR gate and XNOR gate by adding certain cells to the basic building and setting up the customization cells.

The proposed basic building block can also be customized to implement reversible gates such as CNOT gate and Toffoli gate by adding certain cells to the block with appropriate setting of the customization cells. Moreover, it has been shown that the basic building block can implement reversible circuits that contain multiple reversible gates. It has been shown that the QCA that uses the proposed basic building block is more efficient in terms of cell count and/or better clock cycle delay than other proposed designs.

## Figures and Tables

**Table 1:** Customization of the 11 configuration cells *x*_1_, *x*_2_, …, *x*_11_ of the proposed basic building block where DC denotes device cell, OC denotes output cell, IC denotes input cell, and RC denotes rotate cell.

Gates	Configuration cells											Number of added cells
	*x*_1_	*x*_2_	*x*_3_	*x*_4_	*x*_5_	*x*_6_	*x*_7_	*x*_8_	*x*_9_	*x*_10_	*x*_11_	
AND gate	DC	DC	DC	DC	DC	DC	DC	OC	IC	IC	−1.0	0
OR gate	OC	IC	IC	−1.0	DC	DC	DC	DC	DC	DC	DC	0
NOT gate	DC	DC	DC	DC	IC	OC	DC	DC	DC	DC	DC	0
XOR gate	−1.0	DC	1.0	−1.0	IC	DC	DC	DC	1.0	1.0	1.0	1
XNOR gate	−1.0	DC	1.0	−1.0	IC	DC	DC	DC	1.0	1.0	1.0	3
CNOT gate	−1.0	DC	1.0	−1.0	IC	DC	DC	DC	1.0	1.0	1.0	4
Toffoli gate	−1.0	DC	1.0	−1.0	IC	RC	RC	DC	1.0	1.0	1.0	9
Reversible circuit in Figure 14	−1.0	DC	1.0	−1.0	IC	DC	DC	DC	1.0	1.0	1.0	10

**Table 2:** Comparison between the proposed QCA design for the XOR gate and other designs.

XOR logic structures	Complexity (cell count)	MVs	Latency (clocking cycles)
Other designs			
7th design, Fig. 15 in [13]	42	3	1
1st design, Fig. 9 in [13]	34	4	1
Fig. 9 in [14]	30	3	4
Fig. 5e in [12]	30	3	0.5
The proposed design			
Figure 8	28	2	0

**Table 3:** Comparison between the proposed QCA design for the Toffoli gate and other designs.

Toffoli logic structures	Complexity (cell count)	Latency (clock cycle)
Other designs		
Fig. 5 in [2]	169	4
Fig. 7b in [15]	75	1
Fig. 7a in [15]	48	1
Fig. 4.5 in [16]	44	1
The proposed design		
Figure 13	36	2
